# Mechanisms and applications of the anti-inflammatory effects of photobiomodulation

**DOI:** 10.3934/biophy.2017.3.337

**Published:** 2017-05-19

**Authors:** Michael R Hamblin

**Affiliations:** 1 Wellman Center for Photomedicine, Massachusetts General Hospital, BAR414, 40 Blossom Street, Boston, MA 02114, USA; 2 Department of Dermatology, Harvard Medical School, Boston, MA 02115, USA; 3 Harvard-MIT Division of Health Sciences and Technology, Cambridge, MA 02139, USA

**Keywords:** photobiomodulation, low-level laser therapy, chromophores, inflammation, animal studies, clinical trials

## Abstract

Photobiomodulation (PBM) also known as low-level level laser therapy is the use of red and near-infrared light to stimulate healing, relieve pain, and reduce inflammation. The primary chromophores have been identified as cytochrome c oxidase in mitochondria, and calcium ion channels (possibly mediated by light absorption by opsins). Secondary effects of photon absorption include increases in ATP, a brief burst of reactive oxygen species, an increase in nitric oxide, and modulation of calcium levels. Tertiary effects include activation of a wide range of transcription factors leading to improved cell survival, increased proliferation and migration, and new protein synthesis. There is a pronounced biphasic dose response whereby low levels of light have stimulating effects, while high levels of light have inhibitory effects. It has been found that PBM can produce ROS in normal cells, but when used in oxidatively stressed cells or in animal models of disease, ROS levels are lowered. PBM is able to up-regulate anti-oxidant defenses and reduce oxidative stress. It was shown that PBM can activate NF-kB in normal quiescent cells, however in activated inflammatory cells, inflammatory markers were decreased. One of the most reproducible effects of PBM is an overall reduction in inflammation, which is particularly important for disorders of the joints, traumatic injuries, lung disorders, and in the brain. PBM has been shown to reduce markers of M1 phenotype in activated macrophages. Many reports have shown reductions in reactive nitrogen species and prostaglandins in various animal models. PBM can reduce inflammation in the brain, abdominal fat, wounds, lungs, spinal cord.

## 1. Introduction

Photobiomodulation (PBM) was discovered almost 50 years ago by Endre Mester in Hungary [1]. For most of this time PBM was known as “low-level laser therapy” as ruby laser (694 nm) and HeNe lasers (633 nm) were the first devices used. Recently a consensus decision [2] was taken to use the terminology “PBM” since the term “low-level” was very subjective, and it is now known that actual lasers are not required, as non-coherent light-emitting diodes (LEDs) work equally well [3]. For much of this time the mechanism of action of PBM was unclear, but in recent years much progress has been made in elucidating chromophores and signaling pathways [4].

Most of the early work in this field was carried out with various kinds of lasers, and it was thought that laser light had some special characteristics not possessed by light from other light sources such as sunlight, fluorescent or incandescent lamps and now LEDs. However all the studies that have been done comparing lasers to equivalent light sources with similar wavelength and power density of their emission, have found essentially no difference between them.

Many wavelengths in the red (600–700 nm) and near-infrared (NIR, 770–1200 nm) spectral regions have shown positive results, however there is a region in between (700–770 nm) where broadly speaking, the results are likely to be disappointing. Recently blue and green wavelengths have also begun to be explored [5] but they have major problems with penetration depth. It is accepted that penetration of light into tissue is governed by both absorption and scattering by molecules and structures present in tissue. Both absorption and scattering become significantly less as the wavelength gets longer, so the penetration depth of NIR is maximal about 810 nm, and at longer wavelengths water becomes an important absorber and penetration depth gets shorter again [6].

The “biphasic dose response” describes a situation in which there is an optimum value of the “dose” of PBM most often defined by the energy density (J/cm^2^) [7,8]. It has been consistently found that when the dose of PBM is increased a maximum response is reached at some value, and if the dose in increased beyond that maximal value, the response diminishes, disappears and it is even possible that negative or inhibitory effects are produced at very high fluences.

## 2. Chromophores Responsible for Photobiomodulation

### 2.1. Cytochrome c oxidase in mitochondria

Cytochrome c oxidase (CCO) is unit IV in the mitochondrial electron transport chain. It transfers one electron (from each of four cytochrome c molecules), to a single oxygen molecule, producing two molecules of water. At the same time the four protons required, are translocated across the mitochondrial membrane, producing a proton gradient that the ATP synthase enzyme needs to synthesize ATP. CCO has two heme centers (a and a3) and two copper centers (Cu_A_ and Cu_B_). Each of these metal centers can exist in an oxidized or a reduced state, and these have different absorption spectra, meaning CCO can absorb light well into the NIR region (up to 950 nm) [9]. Tiina Karu from Russia was the first to suggest [10,11], that the action spectrum of PBM effects matched the absorption spectrum of CCO, and this observation was confirmed by Wong-Riley et al in Wisconsin [12]. The assumption that CCO is a main target of PBM also explains the wide use of red/NIR wavelengths as these longer wavelengths have much better tissue penetration than say blue or green light which are better absorbed by hemoglobin. The most popular theory to explain exactly why photon absorption by CCO could led to increase of the enzyme activity, increased oxygen consumption, and increased ATP production is based on photodissociation of inhibitory nitric oxide (NO) [13]. Since NO is non-covalently bound to the heme and Cu centers and competitively blocks oxygen at a ratio of 1:10, a relatively low energy photon can kick out the NO and allow a lot of respiration to take place [14].

### 2.2. Light gated ion channels and opsins

More recently it has become apparent that another class of photoreceptors, must be involved in transducing cellular signals, particularly responding to blue and green light. Thee photoreceptors have been proposed to be members of the family of light-sensitive G-protein coupled receptors known as opsins (OPN). Opsins function by photoisomerization of a cis-retinal co-factor leading to a conformational change in the protein. The most well known opsin is rhodopsin (OPN1), which is responsible for mediating vision in the rod and cone photoreceptor cells in the mammalian retina. There are other members of the opsin family (OPN2-5), which are expressed in many other tissues of the body including the brain [15]. One of the best-defined signaling events that occurs after light-activation of opsins, is the opening of light-gated ion channels such as members of the transient receptor potential (TRP) family of calcium channels [16]. TRP channels are now known to be pleiotropic cellular sensors mediating the response to a wide range of external stimuli (heat, cold, pressure, taste, smell), and involved in many different cellular processes [17]. Activation of TRP causes non-selective permeabilization (mainly of the plasma membrane) to calcium, sodium and magnesium [18]. It is now known that TRP channel proteins are conserved throughout evolution and are found in most organisms, tissues, and cell-types. The TRP channel superfamily is now classified into seven related subfamilies: TRPC, TRPM, TRPV, TRPA, TRPP, TRPML, and TRPN [19]. Light-sensitive ion channels are based on an opsin chromophore (isomerization of a cis-retinal molecule to the trans configuration) as illustrated in *Drusophila* photoreceptors [20].

We have shown that blue or green light (but not red or 810 nm NIR) increased intracellular calcium in adipose derived stem cells, that could be blocked by ion channel inhibitors [5].

### 2.3. Flavins and flavoproteins

There is another well-known family of biological chromophores called cryptochromes. These proteins have some sequence similarity to photolyases [21], which are blue light responsive enzymes that repair DNA damage in bacteria caused by UV exposure [22]. Cryptochromes rely on a flavin (flavin adenine dinucleotide, FAD) or a pterin (5,10-methenyltetrahydrofolic acid) to actually absorb the light (again usually blue or green). Cryptochromes have been studied mainly in plants and insects. Recent evidence has emerged that mammalian cryptochromes are important in regulation of the circadian clock. It is thought that human cryptochromes (CRY1 and CRY2) send signals via part of the optic nerve to the suprachiasmatic nucleus (SCN) in the brain, which is the master regulator of the CLOCK system to entrain biological responses to the light-dark cycle [23]. However the situation is complicated because retinal ganglion cells containing melanopsin (OPN4) are also involved in photoentrainment [24]. Studies are still ongoing to investigate this redundancy [25].

It should be emphasized that compared to CCO and mitochondria, evidence is still emerging concerning the extent to which opsins, cryptochomes and light-gated ion channels (which may be widely expressed in many different cell types) could be responsible for PBM effects. If their role is significant it is likely to be in the blue and green spectral regions. Further research will be necessary to explore their role in anti-inflammatory effects, wound healing and tissue regeneration.

### 2.4. Water as a chromophore and heat-gated ion channels

Since the biological effects of light continue to be observed, as the wavelength increases in the infra-red region (>1000 nm), beyond those known to be absorbed by CCO, it is now thought likely that an alternative chromophore must be responsible. The obvious candidate for this alternative chromophore is water molecules whose absorption spectrum has peaks at 980 nm, and also at most wavelengths longer than 1200 nm. Moreover, water is by the far the most prevalent molecule in biological tissue (particularly considering its low molecule weight = 18). At present the proposed mechanism involves selective absorption of IR photons by structured water layers (also known as interfacial water) [26] or water clusters [27], at power levels that are insufficient to cause any detectable bulk-heating of the tissue. A small increase in vibrational energy by a water cluster formed in or on a sensitive protein such as a heat-gated ion channel, could be sufficient to perturb the tertiary protein structure thus opening the channel and allowing modulation of intracellular calcium levels [28]. Pollack has shown that interfacial water can undergo charge separation when it absorbs visible or NIR light [29]. This charge separation (equivalent to localized pH changes) could affect the conformation of proteins [30]. It has also been suggested that PBM could reduce the viscosity of interfacial water within the mitochondria, and allow the F_0_F_1_ ATP synthase, which rotates as a nanomotor to turn faster [31]. It should be noted here that the first regulatory approvals of PBM were gained as a 510 K device “equivalent to an non-heating IR lamp” [32]. While the involvement of water as a chromophore may still be considered hypothetical it is difficult to think of another explanation for the beneficial of PBM at wavelengths between 1000 nm all the way to 10,000 nm (carbon dioxide laser).

**Figure 1:**
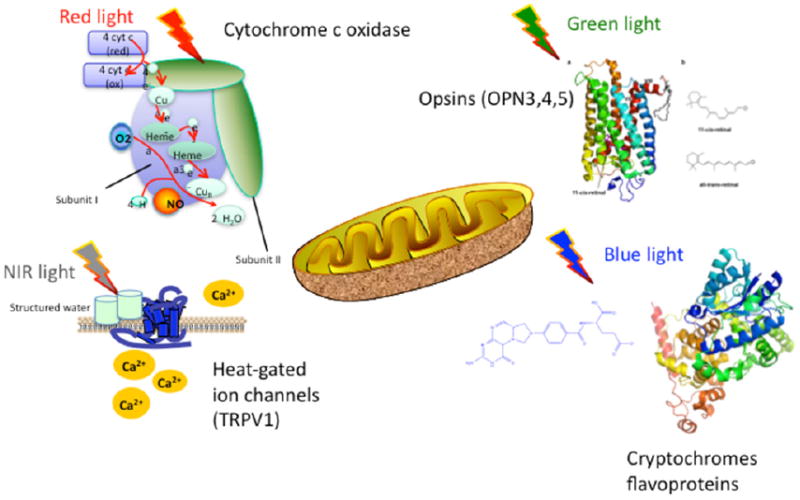
Chromophores in PBM. Cytochrome c oxidase in respiratory chain absorbs mainly red (and NIR) light by heme and copper; Heat-gated TRP ion channels absorb NIR (and blue light) via structured water; opsins absorb mainly blue/green light via cis-retinal; flavoproteins and cryptochromes absorb mainly blue light via pterin.

The molecular chromophores discussed above are graphically summarized in Figure 1.

## 3. Effects of PBM on Reactive Oxygen Species and Oxidative Stress

### 3.1. PBM increases ROS in normal cells

**Figure 2:**
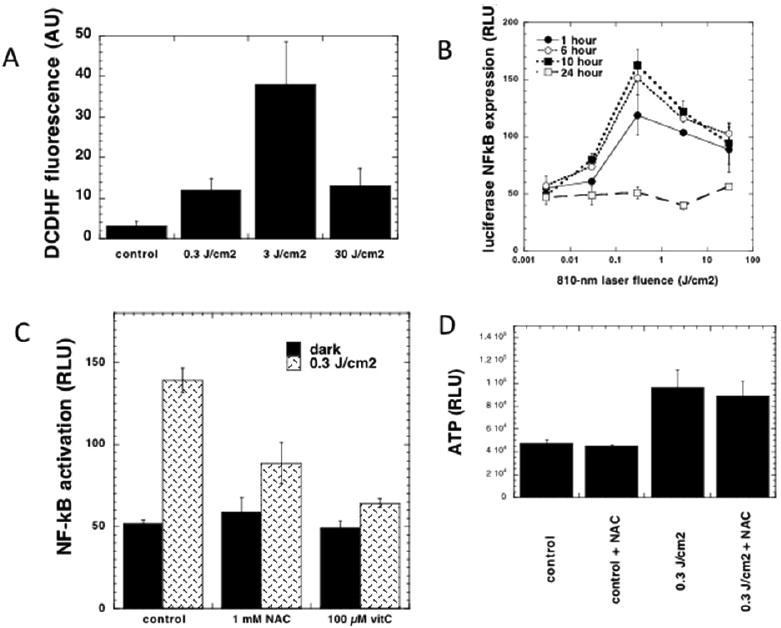
NFkB is activated by PBM induced ROS in embryonic fibroblasts. (A) Intracellular ROS measured by DCDHF fluorescence; (B) NF-kB activation measured by a luciferase assay; (C) NF-kB activation is inhibited by antioxidants; (D) ATP increase is not affected by antioxidants. Figure adapted from data in [34].

**Figure 3:**
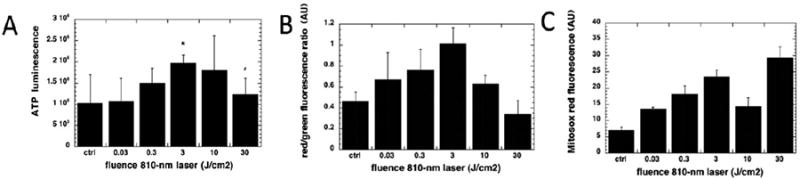
Dose response of 810 nm laser in cortical neurons. (A) ATP production as a function of fluence; (B) Mitochondrial membrane potential (JC1 red/green ratio); (C) Mitochondrial ROS. Figure adapted from data in [35].

When PBM stimulates CCO activity in normal healthy cells, the resulting increase in mitochondrial membrane potential (MMP) above normal baseline levels, leads to a brief and rather modest increase in generation of reactive oxygen species (ROS) [33]. However this brief burst of ROS caused by 3 J/cm^2^ of 810 nm laser (Figure 2A) was shown to be sufficient to activate the redox-sensitive transcription factor, NF-kB in embryonic fibroblasts [34] (Figure 2B). Addition of the anti-oxidant N-acetyl-cysteine to the cells could block the NK-kB activation (Figure 2C), but not the increase in cellular ATP caused by the mitochondrial stimulation (Figure 2D). In primary cultured cortical neurons [35], 810 nm laser produced a biphasic dose response in ATP production (Figure 3A) and MMP (Figure 3B) with a maximum at 3 J/cm^2^. At a high dose (30 J/cm^2^) the MMP was actually lowered below baseline. Interestingly the dose-response curve between fluence (J/cm^2^) and ROS production showed two different maxima (Figure 3C). One of these maxima occurred at 3 J/cm^2^ where the MMP showed its maximum increase. The second maximum in ROS production occurred at 30 J/cm^2^ where the MMP had been reduced below baseline. At a value between these two fluences (10 J/cm^2^) a dose at which the MMP was approximately back to baseline, there was not much ROS generation. These data are very good examples of the “biphasic dose response” or “Arndt-Schulz curve” which is often discussed in the PBM literature [7,8].

Thus it appears that ROS can be generated within mitochondria when the MMP is increased above normal values and also when it is decreased below normal values. It remains to be seen whether these two kinds of PBM-generated ROS are identical or not. One intriguing possibility is that whether the ROS generated by PBM is beneficial or detrimental may depend on the rate at which it is generated. If superoxide is generated in mitochondria at a rate that allows superoxide dismutase (SOD) to detoxify it to hydrogen peroxide, then the uncharged H_2_O_2_ can diffuse out of the mitochondria to activate beneficial signaling pathways, while if superoxide is generated at a rate or at levels beyond the ability of SOD to deal with it, then the charged superoxide may build up inside mitochondria and damage them.

### 3.2. PBM reduces ROS in oxidative stressed cells and tissues

**Figure 4:**
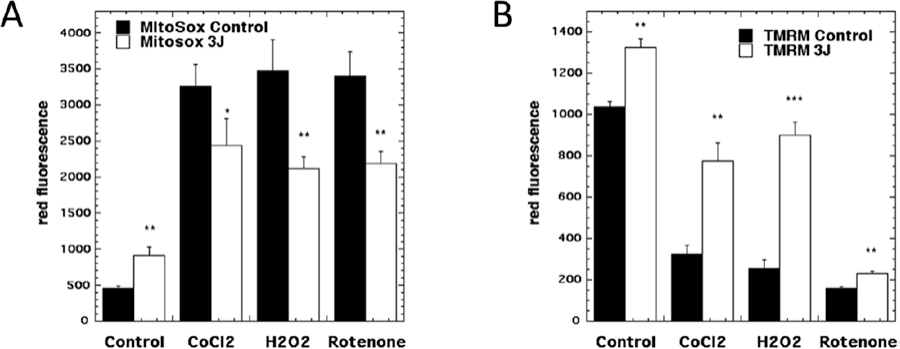
PBM reduces oxidative stress in cortical neurons. Oxidative stress was induced by three different treatments (cobalt chloride, hydrogen peroxide, rotenone) and cells were treated with 3 J/cm^2^ 810 nm. (A) Mitochondrial ROS, (B) Mitochondrial membrane potential (tetramethylrhodamine methyl ester). Figure adapted from data in [39].

Notwithstanding, the ability of PBM to produce a burst of ROS in normal cells, it is well-accepted that PBM when as a treatment for tissue injury or muscle damage is able to reduce markers of oxidative stress [36,37,38]. How can these apparently contradictory findings be reconciled? A study attempted to answer this question [39]. Primary cultured cortical neurons were treated with one of three different interventions, all of which were chosen from literature methods of artificially inducing oxidative stress in cell culture. The first was cobalt chloride (CoCl_2_), which is used as a mimetic for hypoxia and works by a Fenton reaction producing hydroxyl radicals [40]. The second was direct treatment with hydrogen peroxide. The third was treatment with the mitochondrial complex I inhibitor, rotenone [41]. All three of these different treatments increased the intracellular mitochondrial ROS as judged by Cell-Rox Red (Figure 4A), and at the same time lowered the MMP as measured by tetramethyl-rhodamine methyl ester (TMRM) (Figure 4B). PBM (3 J/cm^2^ of 810 nm laser) raised the MMP back towards baseline, while simultaneously reducing the generation of ROS in oxidatively stressed cells (while slightly increasing ROS in normal cells). In control cells (no oxidative stress), PBM increased MMP above baseline and still produced a modest increase in ROS.

Since most laboratory studies of PBM as a therapy have looked at various animal models of disease or injury, it is not surprising that most workers have measured reduction in tissue markers of oxidative stress (TBARS) after PBM [36,42]. There have been a lot of studies looking at muscles. In humans, especially in athletes, high-level exercise produces effects in muscles characterized by delayed-onset muscle soreness, markers of muscle damage (creatine kinase), inflammation and oxidative stress.

One cellular study by Macedo et al [43] used muscle cells isolated from muscular dystrophy mice (mdx LA 24) and found that 5 J/cm^2^ of 830 nm increased the expression levels of myosin heavy chain, and intracellular [Ca^2+^]i. PBM decreased H_2_O_2_ production and 4-HNE levels and also GSH levels and GR and SOD activities. The mdx cells showed significant increase in the TNF-α and NFκB levels, which were reduced by PBM.

While it is highly likely that the effects of PBM in modulating ROS are involved in the anti-inflammatory effects of PBM, it would be dangerous to conclude that that is the only explanation. Other signaling pathways (nitric oxide, cyclic AMP, calcium) are also likely to be involved in reduction of inflammation.

## 4. Effects of PBM on NF-kB

### 4.1. PBM activates NF-kB in normal cells

As mentioned above we found [34] that PBM (3 J/cm^2^ of 810 nm laser) activated NF-kB in embryonic fibroblasts isolated from mice that had been genetically engineered to express firefly luciferase under control of an NF-kB promoter. Although it is well-known that NF-kB functions as a pro-inflammatory transcription factor, but on the other hand it is also well known that in clinical practice or in laboratory animal studies) PBM has a profound anti-inflammatory effect in vivo. This gives rise to another apparent contradiction that must be satisfactorily resolved.

### 4.2. PBM reduces levels of pro-inflammatory cytokines in activated inflammatory cells

**Figure 5:**
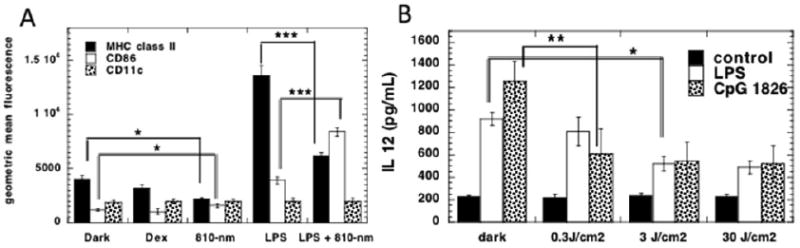
PBM reduces inflammatory markers in activated murine DCs in vitro. (A) Flow cytometry was used to measure MHC class II, CD86, CD11c (dexamethasone was used as positive control); (B) Secreted IL12 measured by ELISA. Figure adapted from data in [44].

Part of the answer to the apparent contradiction highlighted above, was addressed in a subsequent paper [44]. We isolated primary bone marrow-derived dendritic cells (DCs) from the mouse femur and cultured them with GM-CSF. When these cells were activated with the classical toll-like receptor (TLR) agonists, LPS (TLR4) and CpG oligodeoxynucleotide (TLR9), they showed upregulation of cell-surface markers of activation and maturation such as MHC class II, CD86 and CD11c as measured by flow cytometry. Moreover IL12 was secreted by CpG-stimulated DCs. PBM (0.3 or 3 J/cm^2^ of 810 nm laser) reduced all the markers of activation and also the IL12 secretion. Figure 5.

Yamaura et al [45] tested PBM (810 nm, 5 or 25 J/cm^2^) on synoviocytes isolated from rheumatoid arthritis patients. They applied PBM before or after addition of tumor necrosis factor-α (TNF-α). mRNA and protein levels of TNF-α and interleukins (IL)-1beta, and IL-8 were reduced (especially by 25 J/cm^2^).

Hwang et al [46] incubated human annulus fibrosus cells with conditioned medium obtained from macrophages (THP-1 cells) containing proinflammatory cytokines IL1β, IL6, IL8 and TNF-α. They compared 405, 532 and 650 nm at doses up to 1.6 J/cm^2^. They found that all wavelengths reduced IL8 expression and 405 nm also reduced IL6.

The “Super-Lizer” is a Japanese device that emits linear polarized infrared light. Imaoka et al [47] tested it against a rat model of rheumatoid arthritis involving immunizing the rats with bovine type II collagen, after which they develop autoimmune inflammation in multiple joints. The found reductions in IL20 expression in histological sections taken from the PBM-treated joints and also in human rheumatoid fibroblast-like synoviocyte (MH7A) stimulated with IL1β.

Lim et al [48] studied human gingival fibroblasts (HGF) treated with lipopolysaccharides (LPS) isolated from *Porphyromonas gingivalis.* They used PBM mediated by a 635 nm LED and irradiated the cells + LPS directly or indirectly (transferring medium from PBM treated cells to other cells with LPS). Both direct and indirect protocols showed reductions in inflammatory markers (cyclooxygenase-2 (COX2), prostaglandin E2 (PGE2), granulocyte colony-stimulating factor (GCSF), regulated on activated normal T-cell expressed and secreted (RANTES), and CXCL11). In the indirect irradiation group, phosphorylation of C-Raf and Erk1/2 increased. In another study [49] the same group used a similar system (direct PBM on HGF + LPS) and showed that 635 nm PBM reduced IL6, IL8, p38 phosphorylation, and increased JNK phosphorylation. They explained the activation of JNK by the growth promoting effects of PBM. Sakurai et al reported [50] similar findings using HGF treated with *Campylobacter rectus* LPS and PBM (830 nm up to 6.3 J/cm^2^) to reduce levels of COX2 and PGE2. In another study [51] the same group showed a reduction in IL1β in the same system.

### 4.3. Effects of PBM on macrophage phenotype

Another very interesting property of PBM is its ability to change the phenotype of activated cells of the monocyte or macrophage lineage. These cells can display two very different phenotypes depending on which pathological situation the cells are faced with. The M1 phenotype (classically activated) applies to macrophages that are faced with a situation in which bacteria or other pathogens need to be killed, or alternatively tumor cells need to be destroyed. Inducible nitric oxide synthase is a hallmark of the M1 phenotype and nitric oxide secretion is often measured. On the other hand the M2 phenotype (alternatively activated) applies to macrophages that are involved in disposal of cellular or protein debris and stimulation of healing by angiogenesis. The M2 phenotype produces arginase, an enzyme that inhibits NO production and allows them to produce ornithine, a precursor of hydroxyproline and polyamines [52]. The markers of these two phenotypes of activated macrophage have some aspects in common, but also show many aspects that are very different [53]. It should be noted that this concept of M1 and M2 activation states, applies to other specialized macrophage type cells that are resident in different tissues, such as microglia in brain [54], alveolar macrophages in lung [55], Kuppfer cells in liver [56], etc.

Fernandes et al used J774 macrophage-like cells activated with interferon-γ and LPS to produce a MI phenotype and compared 660 nm and 780 nm laser. They found that both wavelengths reduced TNF-α, COX-2 and iNOS expression, with the 780 nm being somewhat better [57]. Silva et al used RAW264.7 macrophages to test two wavelengths (660 nm and 808 nm) at a range of fluences (11-214 J/cm^2^) [58]. They found increases in NO release with 660 nm at the higher fluences. von Leden et al carried out an interesting study looking at the effects of PBM on microglia and their interaction with cortical neurons [59]. They used both primary microglia isolated from mouse brains and the BV2 mouse microglial cell line and compared four fluences (0.2, 4, 10, and 30 J/cm^2^, at 808 nm. Fluences between 4 and 30 J/cm^2^ induced expression of M1 markers in microglia. Markers of the M2 phenotype, including CD206 and TIMP1, were observed at lower energy densities of 0.2–10 J/cm^2^. In addition, co-culture of PBM or control-treated microglia with primary neuronal cultures demonstrated a dose-dependent effect of PBM on microglial-induced neuronal growth and neurite extension. This suggests that the benefits of PBM on neuroinflammation may be more pronounced at lower overall doses. The same group went on to show that M1 activated macrophages receiving PBM (660 nm laser) showed significant decreases in CCL3, CXCL2 and TNFα mRNA expression 4 h after irradiation [60]. However, 24 h after irradiation, M1 macrophages showed increased expression of CXCL2 and TNFα genes. M1 activated macrophages irradiated with 780 nm showed a significant decrease in CCL3 gene expression 4h after irradiation. These data could explain the anti-inflammatory effects of LLLT in wound repair.

## 5. Effects of PBM on Inflammation in Animal Models of Disease

**Figure 6:**
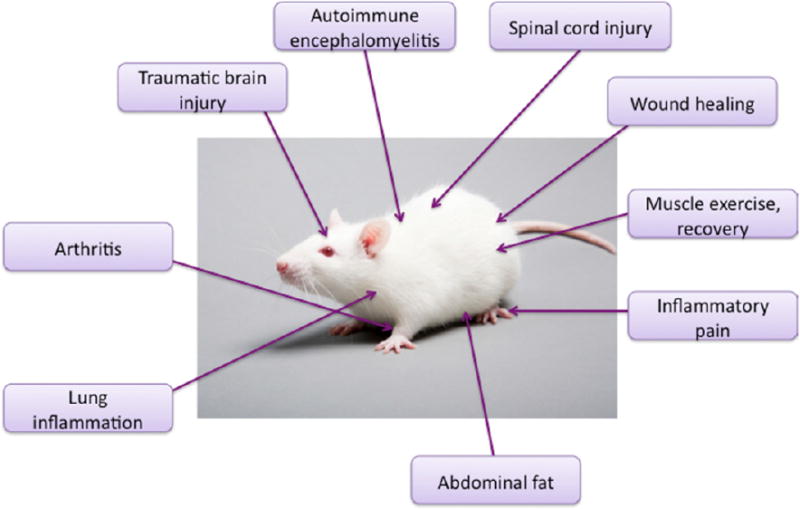
Animal models in which the anti-inflammatory effects of PBM have been shown. Acute traumatic brain injury; experimental autoimmune encephalomyelitis; spinal cord injury; wound healing; muscle exercise and recovery; inflammatory pain in paw; abdominal fat; lung inflammation; knee arthritis.

This section will cover some of the most important medical indications where PBM has been shown in laboratory studies to be effective (at least partly) by its pronounced anti-inflammatory effects. Figure 6 shows a graphical summary of the anti-inflammatory applications of PBM in experimental animal models.

### 5.1. Wound healing

Many papers have demonstrated the efficacy of PBM in stimulating wound healing. In animal models these studies have generally been on acute wounds [61], while in clinical trials they are often been concerned with chronic non-healing wounds such as diabetic ulcers [62]. Gupta et al [63] tested PBM using a superpulsed 904 nm laser on burn wounds in rats. They found faster healing, reduced inflammation (histology), decreased expression of TNF-α and NF-kB, and up-regulated expression of VEGF, FGFR-1, HSP-60, HSP-90, HIF-1α and matrix metalloproteinases-2 and 9 compared to controls. It is intriguing to speculate that the effects of PBM on wound healing (especially the use of for chronic non-healing wounds) could involve both pro-inflammatory effects and anti-inflammatory effects. This seemingly contradictory statement may be possible due to the recent discovery of resolvins and protectins, which are multifunctional lipid mediators derived from omega-3 polyunsaturated fatty acids [64]. If resolvins were produced as a result of the brief acute inflammation induced by application of PBM to chronic wounds, then it has been already shown that resolvins can hasten the healing of diabetic wounds in mice [65]. Resolvins have been shown to reduce tumor necrosis factor-α, interleukin-1β, and neutrophil platelet-endothelial cell adhesion molecule-1 in a mouse burn wound model [66].

### 5.2. Arthritis

In humans, arthritis is most often caused by a degenerative process occurring in osteoarthritis, or an autoimmune process occurring in rheumatoid arthritis. Both are characterized by pronounced inflammatory changes in the joint and even systemically. Different animal models are produced to mimic these diseases, but a common approach is to inject the sterile preparation of yeast cell walls known as zymosan into the knee joints of rats.

Castano et al [67] used this zymosan-induced arthritis model to study the effects of two different fluences of 810 nm laser (3 and 30 J/cm^2^) delivered at two different power densities (5 and 50 mW/cm^2^). PBM was delivered once a day for 5 days commencing after zymosan injection, and the swelling in the knee was measured daily. Prostagladin E2 (PGE2) was measured in the serum. They found that 3 out of the 4 sets of parameters were approximately equally effective in reducing swelling and PGE2, but the ineffective set of parameters was 3 J/cm^2^ delivered at 50 mW/cm^2^ which only took 1 min of illumination time. The conclusion was, that the illumination time was important in PBM, and if that time was too short, then the treatment could be ineffective.

Moriyama et al [68] used a transgenic mouse strain (FVB/N-Tg(iNOS-luc) that had been engineered to express luciferase under control of the inducible nitric oxide synthase promoter, to allow bioluminescence imaging of PBM of the zymosal-induced arthritis model in mice knees. They compared the same fluence of 635, 660, 690, and 905 nm (CW0 and 905 nm (short pulse). Animals younger than 15 weeks showed mostly reduction of iNOS expression, while older animals showed increased iNOS expression. Pulsed 905 nm also increased iNOS expression.

Pallotta et al [69] used a model where carageenan was injected into the rat knee and tested 810 nm laser at 1, 3, 6 or 10 J/cm^2^. Rats were sacrificed after 6 or 12 hours and the joint tissue removed. PBM was able to significantly inhibit the total number of leukocytes, as well as the myeloperoxidase activity. Vascular extravasation was significantly inhibited at the higher dose of energy of 10 J. Gene expression of both COX-1 and 2 were significantly enhanced by laser irradiation while PGE2 production was inhibited. These apparently contradictory results require more study to fully explain.

### 5.3. Muscles

One of the most robust applications of PBM, is its effects on muscles [70,71]. PBM can potentiate muscular performance especially when applied to the muscles 3 hours before exercise [72]. PBM can also make exercise-training regimens more effective. It is not therefore surprising that PBM can also help to heal muscle injuries, not to mention reducing muscle pain and soreness after excessive exercise. Many of the animal studies that have been done have looked at markers of inflammation and oxidative stress in muscle tissue removed from sacrificed animals. For instance, Silveira et al [73] caused a traumatic muscle injury by a single blunt-impact to the rat gastrocnemius muscle. PBM (850 nm, 3 or 5 J/cm^2^) was initiated 2, 12, and 24  h after muscle trauma, and repeated for five days. The locomotion and muscle function was improved by PBM. TBARS, protein carbonyls, superoxide dismutase, glutathione peroxidase, and catalase, were increased after muscle injury, these increases were prevented by PBM. PBM prevented increases in IL-6 and IL-10 and reversed the trauma-induced reduction in BDNF and VEGF.

### 5.4. Inflammatory pain

There have been many studies that have looked at the effects of PBM on pain in animal models. Some studies have looked at sensitivity to pain [74] using the von Frey filaments (a graded set of fibers of increasing stiffness and when the animal feels the pressure it withdraws its foot [75]).

Some studies have looked at animal models of neuropathic pain such as the “spared nerve injury” [76]. This involves ligating two out of three branches of the sciatic nerve in rats and causes long lasting (>6 months) mechanical allodynia [77]. Kobelia Ketz et al found improvements in pain scores with PBM (980  nm applied to affected hind paw 1 W, 20 s, 41 cm above skin, power density 43.25  mW/cm^2^, dose 20 J). They also found lower expression of the proinflammatory marker (Iba1) in microglia in the dorsal root ganglion, gracile nucleus, dorsal column and dorsal horn. The M1/M2 balance of the macrophage phenotype was switched from M1 to M2 by PBM, as judged by relative staining with anti-CD86 (M1) and anti-CD206 (M2).

Martins et al looked at the effect of PBM on a model of inflammatory pain [42]. This involved injecting complete Freund's adjuvant (CFA) into the mouse paw, and produces hyperalgesia and elevated cytokine levels (TNF-α, IL-1β, IL-10). They found that LEDT (950-nm, 80 mW/cm^2^, 1, 2 or 4 J/cm^2^) applied to the plantar aspect of the right hind limb, reduced pain, increased the levels of IL-10 prevented TBARS increase in both acute and chronic phases, reduced protein carbonyl levels and increased SOD and CAT activity in the acute phase only.

### 5.5. Lung inflammation

Aimbire and his laboratory in Brazil have carried out several studies on the use of PBM to reduce acute lung inflammation (ALI) in various animal models. In a mouse model of lung inflammation caused either by inhalation of lipolysaccharide or intranasal administration of TNFα they analyzed the bronchoalveolar lavage fluid (BALF). PBM (660 nm, 4.5 J/cm^2^) was administered to the skin over the right upper bronchus 15 min after ALI induction. PBM attenuated the neutrophil influx and lowered TNFα in BALF. In alveolar macrophages, PBM increased cAMP and reduced TNFα mRNA.

They also studied a different model of ALI caused by intestinal ischemia and reperfusion (I/R), that produces an analogue of acute respiratory distress syndrome (ARDS) [78]. Rats were subjected to superior mesenteric artery occlusion (45 min) and received PBM (660 nm, 7.5 J/cm^2^) carried out by irradiating the rats on the skin over the right upper bronchus for 15 and 30 min, and rats were euthanized 30 min, 2, or 4 h later. PBM reduced lung edema, myeloperoxisdase activity, TNF-α and iNOS, LLLT increased IL-10 in the lungs of animals subjected to I/R.

A third animal model was related to asthma [79]. Mice were sensitized to ovalbumin (OVA), and then challenged by a single 15-min exposure to aerosolized OVA. PBM was applied as above (660 nm, 30 mW, 5.4 J). Bronchial hyper-responsiveness (as measured by dose response curves to acetylcholine) was reduced by PBM as well as reductions in eosinophils and eotaxin. PBM also diminished expression of intracellular adhesion molecule and Th2 cytokines, as well as signal transducer and activator of transduction 6 (STAT6) levels in lungs from challenged mice. Recently Rigonato-Oliveira et al. presented a study that concluded that the reduced lung inflammation and the positive effects of PBM on the airways appear to be mediated by increased secretion of the anti-inflammatory cytokine IL-10, and reduction of mucus in the airway [80].

### 5.6. Traumatic brain injury

In recent years the use of PBM as a treatment for traumatic brain injury [81,82], and other brain disorders including stroke, neurodegenerative diseases and even psychiatric disorders has increased markedly [83]. It is thought that the actions of NIR light shone on the head and penetrating into the brain are multi-factorial, but one clear effect is the anti-inflammatory action of transcranial PBM. This was shown by a series of mouse experiments conducted by Khuman et al [84]. They used the controlled cortical impact model of TBI and delivered PBM (800  nm) was applied directly to the contused parenchyma or transcranially in mice beginning 60–80 min after CCI. Injured mice treated with 60 J/cm^2^ (500  mW/cm^2^ × 2  min) had improved latency to the hidden platform and probe trial performance in the Morris water maze. PBM in open craniotomy mice reduced the number of activated microglia in the brain at 48  h (21.8 ± 2.3 versus 39.2 ± 4.2 IbA-1 + cells/field).

### 5.7. Spinal cord injury

Spinal cord injury (SCI) is another promising area of central nervous system injury that could be benefited by PBM. Veronez et al [85] used a rat model of SCI involving a contusion produced by a mechanical impactor (between the ninth and tenth thoracic vertebrae), with a pressure of 150 kdyn. Three different doses of PBM (808-nm laser) were tested: 500 J/cm^2^, 750 J/cm^2^ and 1000 J/cm^2^ delivered daily for seven days. Functional preformance and tactile sensitivity were improved after PBM, at 1000 J/cm^2^. PBM at 750 and 1000 J/cm^2^ reduced the lesion volume and also reduced markers of inflammation (lower CD-68 protein expression).

### 5.8. Autoimmune diseases

Experimental autoimmune encephalomyelitis (EAE) is the most commonly studied animal model of multiple sclerosis (MS), a chronic autoimmune demyelinating disorder of the central nervous system. Immunomodulatory and immunosuppressive therapies currently approved for the treatment of MS slow disease progression, but do not prevent it. Lyons et al [86] studied a mouse model of EAE involving immunization with myelin oligodendrocyte glycoprotein (MOG35-55). They treated the female C57BL/6 mice with PBM (670 nm) for several days in different regimens. In addition to improved muscular function, they found down-regulation of inducible nitric oxide synthase (iNOS) gene expression in the spinal cords of mice as well as an up-regulation of the Bcl-2 anti-apoptosis gene, an increased Bcl-2:Bax ratio, and reduced apoptosis within the spinal cord of animals over the course of disease. 670 nm light therapy failed to ameliorate MOG-induced EAE in mice deficient in iNOS, confirming a role for remediation of nitrosative stress in the amelioration of MOG-induced EAE by 670 nm mediated photobiomodulation.

### 5.9. Abdominal fat

Yoshimura et al [87] looked at a mouse model of obesity and type 2 diabetes [87]. Four weeks old male adult C57BL/6 mice were fed a hypercaloric high-fat diet (40% calories derived from fat) for eight weeks to induce obesity and hyperglycemia. Over a period of four weeks mice were exposed to six irradiation sessions using an 843 nm LED (5.7 J cm^−2^, 19 mW cm^−2^). Non-irradiated control mice had areas of inflammation in their abdominal fat almost five times greater than the PBM group. The PBM group had significantly lower blood glucose levels 24 hours after the last session.

## 6. Clinical Applications of PBM for Inflammation

Amongst the many hundreds of reports of clinical applications of PBMT, we will highlight a few here, which seem to be especially relevant to inflammation, and inflammatory disorders.

### 6.1. Achilles tendinopathy

Bjordal et al in Norway carried out a randomized, placebo controlled trial of PBM (904 nm, 5.4 J per point, 20 mW/cm^2^) for activated Achilles tendinitis [88]. In addition to clinical assessment, they used microdialysis measurement of peritendinous prostaglandin E2 concentrations. Doppler ultrasonography measurements at baseline showed minor inflammation shown by increased intratendinous blood flow, and a measurable resistive index. PGE2 concentrations were significantly reduced with PBM vs placebo. The pressure pain threshold also increased significantly.

### 6.2. Thyroiditis

Chavantes and Chammas in Brazil have studied PBM for chronic autoimmune thyroiditis. An initial pilot trial [89] used 10 applications of PBM (830 nm, 50 mW, 38–108 J/cm^2^), twice a week, using either the punctual technique (8 patients) or the sweep technique (7 patients). Patients required a lower dosage of levothyroxine, and showed an increased echogenicity by ultrasound. The next study [90] was a randomized, placebo-controlled trial of 43 patients with a 9-month follow-up. In addition to improved thyroid function they found reduced autoimmunity evidenced by lower thyroid peroxidase antibodies (TPOAb), and thyroglobulin antibodies (TgAb). A third study [91] used color Doppler ultrasound to show improved normal vascualrization in the thyroid parenchyma. Finally [92] they showed a statistically significant increase in serum TGF-β1 levels 30 days post-intervention in the PBM group, thus confirming the anti-inflammatory effect. Recently a long-term follow up study of these thyroiditis patients (6 years later) was presented showing that PBM was safe in the long term and demonstrated lasting benefits [93].

### 6.3. Muscles

PBM for muscles aims to benefit athletic performance and training, to reduce delayed onset muscle soreness (DOMS), as well as to ameliorate signs of muscle damage (creatine kinase) after intense or prolonged exercise. Moreover PBM can also be used to treat frank muscle damage caused by muscle strains or trauma. The International Olympic Committee and the World Anti-Doping Agency cannot ban light therapy for athletes considering (1) the intensity is similar to sunlight, and (2) there is no forensic test for light exposure. There have been several clinical trials carried out in Brazil in athletes such as elite runners [94], volleyball players [95] and rugby players [96]. Ferraresi et al conducted a case-controlled study in a pair of identical twins [97]. They used a flexible LED array (850 nm, 75 J, 15 sec) applied to both quadriceps femoris muscles (real to one twin and sham to the other) immediately after each strength training session (3 times/wk for 12 weeks) consisting of leg press and leg extension exercises with load of 80% and 50% of the 1-repetition maximum test, respectively. PBM increased the maximal load in exercise and reduced fatigue, creatine kinase, and visual analog scale (DOMS) compared to sham. Muscle biopsies were taken before and after the training program and showed that PBM decreased inflammatory markers such as interleukin 1β and muscle atrophy (myostatin). Protein synthesis (mammalian target of rapamycin) and oxidative stress defense (SOD2, mitochondrial superoxide dismutase) were up-regulated.

### 6.4. Psoriasis

Psoriasis is a chronic autoimmune skin disease. Psoriasis is characterized by the abnormally excessive and rapid growth of keratinocytes (instead of being replaced every 28–30 days as in normal skin, in psoriatic skin they are replaced every 3–5 days). This hyperproliferation is caused by an inflammatory cascade in the dermis involving dendritic cells, macrophages, and T cells secreting TNF-α, IL-1β, IL-6, IL-17, IL-22, and IL-36γ [98]. PBM has been used for psoriasis because of its anti-inflammatory effects, which is a different approach from UV phototherapy which tends to kill circulating T-cells. Ablon [99] tested PBM using LEDs (830 nm, 60 J/cm^2^ and 633 nm, 126 J/cm^2^) in two 20-min sessions over 4 or 5 weeks, with 48 h between sessions in 9 patients with chronic treatment-resistant psoriasis. Clearance rates at the end of the follow-up period ranged from 60% to 100%. Satisfaction was universally very high.

Choi et al [100] tested PBM in case report of a patient with another inflammatory skin disease called acrodermatitis continua, who also had a 10-yr history of plaque psoriasis on her knees and elbows. As she was pregnant and not suited for pharmacological therapy, she received treatment with PBM (broad-band polarized light, 480–3,400 nm, 10 J/cm^2^). In two weeks (after only 4 treatments), the clinical resolution was impressive and no pustules were found. Topical methylprednisolone aceponate steroid cream was switched to a moisturizer, and she was treated twice or once a week with PBM until a healthy baby was delivered.

### 6.5. Arthritis

As can be seen from the animal studies section, arthritis is one of the most important clinical indications for PBM [101,102]. The two most common forms of arthritis are osteoarthritis (degenerative joint disease that mostly affects the fingers, knees, and hips) and rheumatoid arthritis (autoimmune joint inflammation that often affects the hands and feet). Osteoarthritis (OA) affects more than 3.8% of the population while rheumatoid arthritis (RA) affects about 0.24%. Both types have been successfully treated with PBM. Cochrane systematic reviews found for good evidence for its effectiveness in RA [103], and some evidence in the case of OA [104]. Most clinical studies have used pain scales and range of movement scores to test the effectiveness, rather than measures of inflammation which are difficult to carry out in human subjects.

Barabas and coworkers [105] made an attempt by testing PBM on ex vivo samples of synovial tissue surgically removed from patients receiving knee joint replacement. Synovial membrane samples received exposure to PBM (810 nm, 448 mW, 25 J/cm^2^, 1 cm^2^ area). PBM caused an increase in mitochondrial heat shock protein 1 60 kD, and decreases in calpain small subunit 1, tubulin alpha-1C, beta 2,vimentin variant 3, annexin A1, annexin A5, cofilin 1,transgelin, and collagen type VI alpha 2 chain precursor all significantly decreased compared to the control

### 6.6. Alopecia areata

Alopecia areata (AA) is one of the three common types of hair loss, the other two being androgenetic alopecia (AGA, male pattern baldness) and chemotherapy induced alopecia. AA is a common autoimmune disease resulting from damage caused to the hair follicles (HFs) by T cells. Evidence of autoantibodies to anagen stage HF structures is found in affected humans and experimental mouse models. Biopsy specimens from affected individuals demonstrate a characteristic peri- and intrafollicular inflammatory infiltrate around anagen-stage HFs consisting of activated CD4 and CD8 T lymphocytes [106]. PBM is an excellent treatment for hair loss in general and AGA in particular [107,108]. Yamazaki et al [109] reported the use of the “Super-Lizer” delivering linear-polarized light between 600–1600 nm at a power of 1.26 W to the areas of hair loss on the scalp (4-s pulses delivered at 1-s intervals for 3 min every 1 or 2 weeks until hair growth was observed). Regrowth of vellus hairs was achieved on more than 50% ofthe involved areas in all 15 cases. The frequency of irradiation until regrowth ranged from one to 14 times and the duration of SL treatment was 2 weeks to 5 months.

## 7. Conclusion and Future Studies

The clinical applications of PBM have been increasing apace in recent years. The recent adoption of inexpensive large area LED arrays, that have replaced costly, small area laser beams with a risk of eye damage, has accelerated this increase in popularity. Advances in understanding of PBM mechanisms of action at a molecular and cellular level, have provided a scientific rationale for its use for multiple diseases. Many patients have become disillusioned with traditional pharmaceutical approaches to a range of chronic conditions, with their accompanying distressing side-effects and have turned to complementary and alternative medicine for more natural remedies. PBM has an almost complete lack of reported adverse effects, provided the parameters are understood at least at a basic level. The remarkable range of medical benefits provided by PBM, has led some to suggest that it may be “too good to be true”. However one of the most general benefits of PBM that has recently emerged, is its pronounced anti-inflammatory effects. While the exact cellular signaling pathways responsible for this anti-inflammatory action are not yet completely understood, it is becoming clear that both local and systemic mechanisms are operating. The local reduction of edema, and reductions in markers of oxidative stress and pro-inflammatory cytokines are well established. However there also appears to be a systemic effect whereby light delivered to the body, can positively benefit distant tissues and organs.

There is a lot of scope for further work on PBM and inflammation. The intriguing benefits of PBM on some autoimmune diseases, suggests that this area may present a fertile area for researchers. There may be some overlap between the ability of PBM to activate and mobilize stem cells and progenitor cells, and its anti-inflammatory action, considering that one of the main benefits of exogenous stem cell therapy has been found to be its anti-inflammatory effect. The versatile benefits of PBM on the brain and the central nervous system, encourages further study of its ability to reduce neuroinflammation. Chronic diseases of the modern age involving systemic inflammation such as type II diabetes, obesity, Alzheimer's disease, cardiovascular disease and endothelial dysfunction are again worth investigating in the context of PBM.

## Figures and Tables

**Figure 7:**
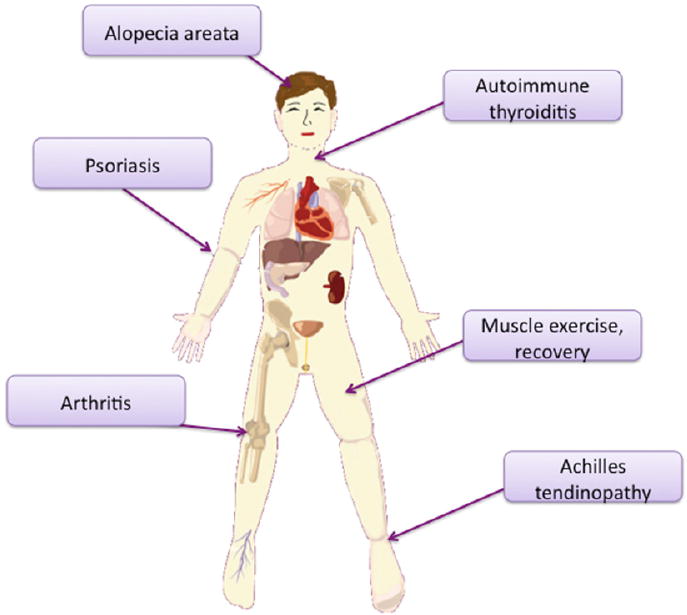
Human clinical indications concentrating on anti-inflammatory effects. Autoimmune thyroiditis; muscle exercise and recovery; Achilles tendinopathy; knee arthritis; psoriasis; alopecia areata.
